# Hemoptysis caused by *Parvimonas micra*: case report and literature review

**DOI:** 10.3389/fpubh.2023.1307902

**Published:** 2024-02-08

**Authors:** Axue Shao, Qingqing He, Xin Jiao, Jianbo Liu

**Affiliations:** ^1^The First Clinical Medical School, Guangzhou University of Chinese Medicine, Guangzhou, China; ^2^The First Affiliated Hospital, Guangzhou University of Chinese Medicine, Guangzhou, China

**Keywords:** *Parvimonas micra*, hemoptysis, case report, literature review, MNGs

## Abstract

**Background:**

*Parvimonas micra* (*P. micra*), a Gram-positive anaerobic bacterium, exhibits colonization tendencies on oral mucosal and skin surfaces, potentially evolving into a pathogenic entity associated with diverse diseases. The diagnostic trajectory for *P. micra*-related diseases encounters delays, often with severe consequences, including fatality, attributed to the absence of symptom specificity and challenges in culture. The absence of a consensus on the diagnostic and therapeutic approaches to *P. micra* exacerbates the complexity of addressing associated conditions. This study aims to elucidate and scrutinize the clinical manifestations linked to *P. micra*, drawing insights from an extensive literature review of pertinent case reports.

**Case presentation:**

A 53-year-old male sought medical attention at our institution presenting with recurrent hemoptysis. Empirical treatment was initiated while awaiting pathogen culture results; however, the patient’s symptoms persisted. Subsequent metagenomic next-generation sequencing (mNGS) analysis revealed a pulmonary infection attributable to *P. micra*. Resolution of symptoms occurred following treatment with piperacillin sulbactam sodium and moxifloxacin hydrochloride. A comprehensive literature review, utilizing the PubMed database, was conducted to assess case reports over the last decade where *P. micra* was identified as the causative agent.

**Conclusion:**

The literature analysis underscores the predilection of *P. micra* for immunocompromised populations afflicted by cardiovascular diseases, diabetes, orthopedic conditions, and tumors. Risk factors, including oral and periodontal hygiene, smoking, and alcohol consumption, were found to be associated with *P. micra* infections. Clinical manifestations encompassed fever, cough, sputum production, and back pain, potentially leading to severe outcomes such as Spondylodiscitis, septic arthritis, lung abscess, bacteremia, sepsis, and mortality. While conventional bacterial culture remains the primary diagnostic tool, emerging technologies like mNGS offer alternative considerations. In terms of treatment modalities, β-lactam antibiotics and nitroimidazoles predominated, exhibiting recovery rates of 56.10% (46/82) and 23.17% (19/82), respectively. This case report and literature review collectively aim to enhance awareness among clinicians and laboratory medicine professionals regarding the intricacies of *P. micra*-associated infections.

## Introduction

*Parvimonas micra*, referred to hereafter as *P. micra*, stands as a Gram-positive anaerobic coccobacillus (GPAC), exhibiting dimensions of approximately 0.3–0.7 μm. Its habitat encompasses the skin and mucosal surfaces of the oral cavity, upper respiratory tract, gastrointestinal tract, and the female genitourinary tract (1, 2). *Parvimonas micra* is implicated as the causative agent in a spectrum of ailments, ranging from sternum osteomyelitis (3), and femur osteomyelitis (4), to sepsis (5), endocarditis (5), hepatic abscesses (6, 7), and even fatal outcomes (1). Notably, *P. micra* predominantly affects immunocompromised hosts (8, 9) with infected individuals often presenting underlying risk factors such as oral infections, diabetes mellitus, and malignant tumors (9, 10). Manifestations of *P. micra* infections typically lack specificity, with reported symptoms encompassing fever, chills, low back pain, abdominal discomfort, gastrointestinal issues, body aches, impaired consciousness, and anorexia in 46 percent of cases (6, 10), as per reviews.

The diagnostic challenge associated with *P. micra* arises from its susceptibility to various pathogens, culture environments, and culture cycles. This often leads to delayed diagnosis, impeding clinicians’ comprehension of the condition, appropriate antibiotic selection, and adversely impacting patient prognosis. To date, literature lacks any documented instances of hemoptysis resulting from *P. micra* infection. In this context, we present a case involving a 53-year-old male with *P. micra* lung infection, aiming to heighten clinicians’ awareness of *P. micra*-induced extraoral infections and recurrent hemoptysis. Our objective is also to investigate the disease characteristics stemming from *P. micra* infection, achieved through a comprehensive literature review of pertinent case reports on *P. micra*. This endeavor seeks to augment awareness of *P. micra* among clinicians and laboratory medicine personnel.

## Case presentation

The patient, a 53-year-old male, sought admission to the First Affiliated Hospital of Guangzhou University of Traditional Chinese Medicine (Guangzhou, China). Over the past year, he reported recurrent hemoptysis episodes, each comprising approximately 30 mL of dark red blood clots, occurring two or three times daily. Notably, the patient exhibited no fatigue or back pain. With a medical history encompassing diabetes mellitus and longstanding tuberculosis, under extended standardized treatment and follow-up for over 30 years, the patient disclosed a smoking history exceeding 10 years. He denied alcohol consumption and reported no family history of infectious or genetic diseases.

Upon admission, the patient presented with a temperature of 36.5°C, a pulse rate of 82 beats/min, a respiratory rate of 20 breaths/min, and a blood pressure reading of 160/95 mm Hg. Physical parameters included a height of 162.0 cm, body weight of 57.50 kg, and a body mass index (BMI) of 21.91 kg/m^2^. Auscultation of respiratory sounds revealed rough sounds in both lungs, with diminished breath sounds in the left upper lung. Initial investigations involved routine cultures of respiratory specimens (sputum culture, *Mycobacterium tuberculosis* sputum smear, etc.), with Supplementary Table 1 displaying the preliminary laboratory results. Notably, the Galactomannan antigen test (GM) and D-glucan fungal antigen test (G test) returned negative results.

Chest CT enhancement images unveiled scattered multiple nodules and mass-like shadows in both lungs, featuring irregularly shaped cavities and necrotic areas (see Supplementary Figure 1). Given these findings and the overall clinical presentation, a high likelihood of pulmonary infection was considered. Consequently, empirical intravenous administration of ceftriaxone sodium (2 g, q12h) was initiated on the first day of admission, pending pathogen identification for tailored antibiotic therapy.

On the fourth day post-admission, sputum smear, routine common bacterial and fungal cultures, and *Mycobacterium tuberculosis* and TB-Dot tests yielded no pathogenic evidence. Fiberoptic bronchoscopy (Supplementary Figure 2) was subsequently performed, obtaining bronchoalveolar lavage fluid (BALF) for routine culture and metagenomic next-generation sequencing (mNGS) analysis at Guangzhou Genguji Laboratory Co (Guangzhou, China). Following standard nucleic acid extraction and library construction procedures, the [NextSeq CN500] platform generated high-quality sequencing data, subsequently compared with the Genghis Khan Pathogenic Microorganisms Database containing 31,000+ microorganism sequences to identify and characterize pathogenic microorganisms (Supplementary Figure 3). BALF yielded 255,421,15 clean sequence readings, of which 190,393 were identified as microorganisms at the genus level, with 583 matching Microcystis at the species level (Supplementary Table 2).

Extended routine culture testing of sputum specimens and BALF samples failed to yield positive results. A thorough patient reexamination, including oral examination, revealed halitosis, dark red, or dark black free gingiva and gingival papilla, easy bleeding on probing, localized tartar or calculus, and some loose and shifted teeth. These oral findings were attributed to the patient’s prolonged site work, lack of dietary attention, poor personal hygiene habits, and chronic smoking. The oral environment corroborated the mNGS results. However, drug sensitivity testing was omitted for this pathogen, given that mNGS routinely tests for drug-resistance genes in anaerobic bacteria only under exceptional circumstances. Subsequent to literature review and consultation with specialists in the Department of Pharmacy, the treatment regimen transitioned to piperacillin sulbactam sodium (6 g, q12h, IV) and moxifloxacin hydrochloride tablets (0.4 g, qd, PO), supplemented with splenic amine oral lyophilized powder (2 mg, qd, PO) and gamma globulin (2.5 g, qd, IV) to modulate the body’s immunity. Simultaneously, pulmonary function rehabilitation training, meridian flow injection, auricular acupuncture, acupoints paste, and other distinctive Chinese medicine treatments were employed to enhance the patient’s disease resistance and promote recovery. Following 7 days of treatment, hemoptysis symptoms abated, leading to the patient’s discharge. Subsequent outpatient follow-up over 2 months indicated an acceptable condition with no hemoptysis recurrence.

## Literature review

### Search strategy

A systematic exploration was conducted on the PubMed online databases employing the keywords: “*Parvimonas micra*,” “Streptococcus micros,” “*Peptostreptococcus micros*,” “Streptococcus anaerobic micros,” “Diplococcus glycinophilus,” and “*Peptococcus glycinophilus*.”

### Inclusion and exclusion criteria

Inclusion criteria encompassed case report studies on *P. micra*-associated diseases (e.g., sepsis, abscesses, and spondylitis) written in English and published after 2013. Exclusion criteria comprised studies published before 2013 and those penned in languages other than English.

### Data extraction and analysis

A meticulous review of all articles was undertaken to eliminate duplicate cases, ensuring comprehensive information for inclusion in the review. Data were meticulously extracted and presented as descriptive statistics in an Excel file.

### Results

#### Search results

A total of 726 documents were retrieved using the designated keywords, yielding 84 case reports. Following a full-text review, 10 articles were excluded (six not published in English, and four not attributing *P. micra* as the primary causative organism), resulting in 74 studies meeting the inclusion criteria. A cumulative 82 patients were included, and the specific clinical characteristics of the 74 studies are detailed in Supplementary Table 3.

#### Demographics

The mean age of patients with *P. micra* was 62.51 years (age range: 23–94 years, excluding one case involving a 9-year-old child). Of the 82 patients, 53 were male (64.63%) and 29 were female (35.37%).

#### Symptom

Prominent complaints included fever (*n* = 23, 28.05%), cough and sputum (*n* = 21, 25.61%), and low back pain (*n* = 18, 21.95%). Conversely, less frequently observed symptoms encompassed sore throat (1, 1.22%) and tachycardia (1, 1.22%).

#### Risk factors/underlying disease

Cardiovascular disease history (e.g., atrial fibrillation, congestive heart failure, and cerebrovascular accident) was observed in 24 (29.27%) patients, hypertension in 19 (23.17%), orthopedic disease in 15 (18.29%), and diabetes mellitus in 14 (17.07%). Additionally, 7 (8.54%) patients had a history of tumors, 19 (23.17%) had oral and periodontal problems, 13 (15.85%) had smoking habits, and six (7.32%) had drinking habits.

#### Infection route

The infection route was reported in 28 (34.15%) cases of oral, 2 (2.44%) cases of the surgical site, 2 (2.44%) cases of mucosal, and 1 (1.22%) case of percutaneous infections. No details about the route of infection were recorded in 49 (59.76%) patients.

#### Diagnosis method

Among patients, 57 (69.51%) used routine bacterial cultures, 22 (26.83%) used blood cultures, and 35 (42.68%) used other tissue cultures such as pus and puncture biopsy tissues. Additionally, nine cases (10.96%) used mNGS testing, nine cases (10.96%) used MALDI-TOF mass spectrometry, and eight cases (9.56%) used 16S rRNA.

#### Organism(s)

Various pathogens associated with concomitant multimicrobial infections included *Streptococcus pyogenes*, *Stenotrophomonas maltophilia*, *Pasteurella multocida*, *Fusobacterium nucleatum*, odontolyticus, *Actinomyces meyeri*, *A. rimae*, and others. *Parvimonas micra* as an individual pathogen was found in 53 cases (64.63%).

#### Desease(s)

In 74 publications, *P. micra* often caused orthopedic-related diseases in 32 cases (39.02%), including spondylodiscitis (11/32, 34.38%), septic arthritis (8/32, 25%); respiratory diseases in 15 cases (18.29%), including lung abscess (7/15, 46.65%), pulmonary embolism (2/15, 13.33%), pneumonia (2/15, 13.33%); and bloodstream infections in 6 cases (7.32%), of which bacteremia (3/6, 50%), sepsis (3/6, 50%).

#### Antibacterial agents

Beta-lactam antibiotics, such as ceftriaxone, piperacillin/tazobactam, and ampicillin-sulbactam, were used in 50 patients; nitazole antibiotics were used in 20 cases; clindamycin was used in 12 cases; and carbapenem antibiotics were used in seven cases. Among them, 34 cases used two or more antibiotics.

#### Outcome

A majority of cases (72/82, 87.80%) were successfully treated, with eight cases (9.76%) admitted to the ICU, of which four cases (9.76%) required mechanical ventilation. Ten cases (12.20%) resulted in mortality due to multiple organ failure. Notably, the recovery rate of patients using β-lactam antibiotics was 56.10% (46/82), with a mortality rate of approximately 4.88%. The cure rate for ceftriaxone was 28% (14/50), piperacillin/tazobactam 14% (7/50), and ampicillin/sulbactam sodium 30% (15/50). For patients with nitroimidazoles, the cure rate was 23.17% (19/82); lincomycin 15.85% (13/82); and carbapenems 3.66% (3/82).

## Discussion

In scrutinizing 82 case reports detailing *P. micra* infections from the past decade, gleaned from PubMed, our analysis reveals a predilection of *P. micra* for instigating orthopedic-related afflictions such as spondylodiscitis, septic arthritis, and post-surgical prosthetic joint infections. Additionally, respiratory disorders, including lung abscess, septic pulmonary embolism, and septic thorax, emerge as significant sequelae. The spectrum extends to rare occurrences of bacteremia and sepsis. This survey underscores intervertebral disks (11), large joints (12), lungs (13), liver (14), and brain (15) as primary sites of *P. micra* invasion, accentuating the imperative consideration of *P. micra* anaerobic infections in tandem with conventional bacterial infections in clinical scenarios involving the aforementioned sites.

Based on the literature review, the initial manifestations of *P. micra* infection encompass fever, cough, sputum production, and lower back pain, alongside additional complaints such as malaise, shortness of breath, and weight loss. Predominantly, patients exhibit fever and pain, with the latter concentrated in the lower back, implicating a potential connection to inflammation induced by *P. micra*. Notably, lipoteichoic acid (LTA) is identified as the virulence factor of *P. micra*, a specialized anaerobic Gram-positive coccobacillus residing in the oral cavity. LTA, a membrane-bound polymer characteristic of Gram-positive bacteria, plays fundamental roles in regulating autolysin activity, scavenging divalent cations (e.g., magnesium ions), and altering the cell wall’s charge properties, thereby influencing bacterial-host cell interactions (16–18).

Recent scholarship, both domestically and internationally, has delved into the biological activity of LTA in Gram-positive pathogenic bacteria within the oral cavity. Findings indicate that LTA elicits immune and inflammatory responses, inducing the release of inflammatory factors like TNF-α, IL-1β, and IL-6 from neutrophils and macrophages, thereby contributing to local inflammation and edema. Moreover, *P. micra* demonstrates robust protein hydrolase activity, generating various pathogenic substances such as protein hydrolase, collagenase, and hyaluronidase (19–21), fostering tissue lysis and inflammation expansion (20, 21).

Some studies reveal that *P. micra*, present in infected root canals of patients with chronic apical periodontitis, engages in metabolic processes utilizing amino acids and polypeptides, establishing trophic symbiosis with dominant pathogenic bacteria in dental pulp (22). This symbiotic relationship enhances mutual virulence and resistance, playing a pivotal role in pathogenic effects. Combining these insights with the literature review, it can be inferred that *P. micra* employs three primary routes to infect different human body parts: bloodstream transmission, direct external inoculation, and transmission from neighboring tissues. Among these, bloodstream dissemination resulting from oral infection emerges as the most prevalent transmission route. Periodontitis history, dental surgery, and poor oral hygiene strongly correlate with *P. micra* infection, as documented in literature (23, 24). Oral infections and compromised oral health facilitate the entry of *P. micra* or soluble toxins into the bloodstream, where they interact with circulating specific antibodies, causing immune damage and inflammation at distant sites (25). Hematogenous dissemination may reach the spine, affecting blood-rich vertebral endplates initially and subsequently leading to local spread to adjacent intervertebral disks or vertebral bodies, resulting in characteristic lesions of intervertebral space infection. Rarely, the infection may spread posteriorly, giving rise to epidural or subdural abscesses, or even meningitis. Lateral spread can lead to various abscess formations, including lumbar major, retroperitoneal, subphrenic, paravertebral, retropharyngeal, and mediastinal abscesses. Primary sites of infection encompass the oral cavity, skin mucosa and soft tissues, respiratory tract, and surgical incisions.

In this presented case, the predominant clinical manifestation was hemoptysis, a recognized symptom frequently associated with pulmonary, bronchial, and circulatory disorders, carrying a substantial risk of asphyxiation and mortality, with an estimated 15% fatality rate in cases of massive hemoptysis (26). Globally, tuberculosis stands as the primary association with hemoptysis; nonetheless, the etiological spectrum encompasses a broad array of pulmonary (e.g., infection, malignancy, airway injury, or trauma), cardiac (e.g., heart failure, cardiac valvular disease, and pulmonary hypertension), vascular (e.g., pulmonary embolism and arteriovenous malformations), vasculitides (e.g., granulomatous polyangiitis), and miscellaneous factors, including coagulation disorders (medical, acquired, and congenital), drug-induced complications, and alveolar protein deposition disorders (27). In the context of our reported case, the patient’s history included remote tuberculosis with hemoptysis over 3 decades prior, which significantly predates the current episode. The patient reported resolution following standardized anti-tuberculosis treatment. Based on the hospitalization, tuberculosis-related test outcomes, and imaging findings, we excluded the recurrence of tuberculosis and bronchiectasis as contributing factors.

Conversely, hemoptysis attributed to *Parvimonas micra* is a clinical rarity. As posited by Dustin L. Higashi, *P. micra* displays characteristic features of an inflammatory organism, thriving in the milieu of active inflammation and the destruction of inflammatory tissue. It ranks among the most prevalent species in numerous studies of mucosal inflammatory diseases and systemic abscesses (28). Murphy’s research further delineates *P. micra*’s induction of macrophages to secrete elevated levels of IL-6, IL-8, and RANTES, along with the stimulation of pro-inflammatory cytokines such as PKA, ERK2, JNK, and p38 from various intracellular signaling pathways. These mechanisms collectively potentiate endotoxin action, thereby influencing the onset of inflammatory responses (2). The hypothesis posits that *P. micra* may release diverse inflammatory mediators through these mechanisms, culminating in the compromise of capillaries within bronchial mucosa or lesions, ultimately resulting in the rupture of submucosal blood vessels and increased permeability, culminating in hemoptysis.

Regarding the diagnostic approaches for *P. micra*, the cases documented in the literature indicate that routine bacterial cultures, including blood culture (26.83%) and culture from puncture biopsy of pus and tissue (42.68%), were predominantly utilized for detection. In our presented case, conventional culture tests yielded negative results, prompting the adoption of metagenomic next-generation sequencing (mNGS) for a definitive diagnosis. This deviation from traditional diagnostic methods is underscored by the inherent limitations of conventional pathogen testing, such as susceptibility to the culture environment, prolonged culture duration, potential impact of prior antibiotic administration, and specific characteristics of the pathogen itself (29). These limitations contribute to the clinical plausibility of false-negative outcomes. Therefore, when conventional pathogen detection yields inconclusive results or when patients exhibit limited improvement despite extended treatment, innovative techniques like mNGS, MALDI-TOF mass spectrometry analysis, and 16S rRNA detection prove invaluable for rapid pathogen identification, guiding subsequent tailored treatments.

Regarding treatment, in this particular case with a clearly identified pathogen, the empirical use of ceftriaxone was replaced with piperacillin sulbactam sodium and moxifloxacin, resulting in alleviation of the patient’s hemoptysis symptoms. It is noteworthy that there is currently no consensus on the antibiotic treatment of *P. micra*. Literature review indicates *P. micra*’s high sensitivity to various antibiotics, including penicillin, meropenem, clindamycin, piperacillin sodium sulbactam sodium, vancomycin, and linezolid (30–32). However, some studies report varying degrees of resistance to penicillin, metronidazole, clindamycin, and vancomycin, while showing susceptibility to imipenem and piperacillin sodium sulbactam sodium (32–34). This aligns with the findings of our case. Notably, our literature review suggests that most cases exhibit favorable outcomes when treated with β-lactams and narrow-spectrum anti-anaerobic drugs like ceftriaxone, piperacillin/tazobactam, ampicillin-sulbactam, clindamycin, and metronidazole. However, our case deviated from this trend, with no improvement observed after ceftriaxone treatment, possibly indicating strain-specific variations in *P. micra*’s response to the same antibiotic (35).

A minority of reported cases (36–38) exhibited uncontrolled infections post-treatment: 8 (9.76%) required ICU admission, 4 (9.76%) underwent mechanical ventilation, and 10 (12.20%) succumbed to multi-organ failure. Consequently, we advocate for drug susceptibility testing specific to *P. micra* to inform antimicrobial usage when conditions permit. Additionally, attention should be directed toward defining the duration of antibiotic use for different sites of *P. micra* infections, and criteria for discontinuation should include symptomatic regression, improvement, and normalization of infection markers such as ESR or CRP. Our case collection underscores the significance of surgery, thorough debridement, and a combination of antimicrobial drugs in addressing *P. micra*-associated abscesses in bone, joints, muscles, lungs, and brain.

In terms of prognosis, 72 patients (87.80%) exhibited a gradual recovery from the infection, while 10 patients (12.20%) succumbed to multiorgan failure (14, 36, 39–44), the majority of whom presented severe comorbid underlying conditions such as cardiovascular diseases, neoplasms, and immune-related diseases. This underscores the imperative need for heightened vigilance regarding *P. micra* infection, particularly in individuals predisposed to compromised oral hygiene, diabetes mellitus, malignancy, and cardiovascular and cerebrovascular diseases. Furthermore, attention should be directed toward patients exhibiting nonspecific symptoms, including high fever of unknown origin, pain, and hemoptysis, with the aim of facilitating early detection and treatment of *P. micra*-induced infections.

In conclusion, we present a case of pulmonary infection attributed to *P. micra*, where hemoptysis serves as the primary symptom. Through a comprehensive review of pertinent literature, our intent is to direct clinicians’ attention toward *P. micra*, offering insights for clinical diagnosis and treatment.

Written informed consent for the publication of this report, inclusive of lung CT images, was obtained from the patients. This study adhered to the principles of the Declaration of Helsinki and received approval from the Research Ethics Committee of the First Affiliated Hospital of Guangzhou University of Traditional Chinese Medicine (JY2022-264).

## Limitation

Nevertheless, several limitations exist in this case report. Firstly, routine culture tests failed to detect *P. micra* and other microorganisms, potentially attributable to various factors such as sampling techniques, professional proficiency, and the purity of *P. micra*. Secondly, alternative diagnostic methods, including 16S rRNA gene sequencing, PCR nucleic acid sequencing, and lung tissue biopsy mNGS, were not employed. Additionally, post-treatment bronchoalveolar lavage fluids were not subjected to mNGS testing to confirm the complete eradication of *P. micra*. Lastly, the mechanism of hemoptysis induced by *P. micra* remains under investigation, and a consensus on the treatment of *P. micra* infection is yet to be established, necessitating further follow-up studies.

## Data availability statement

The raw data has been uploaded and is available in the EBI Metagenomics database with the serial number: PRJEB72347. Further inquiries can be directed to the corresponding author.

## Ethics statement

The studies involving human participants were reviewed and approved by Medicine Ethics Committee of First Affiliated Hospital of Guangzhou University of Chinese Medicine. Written informed consent was obtained from the individual for the publication of any potentially identifiable images or data included in this article.

## Author contributions

AS: Writing – original draft. QH: Writing – original draft. XJ: Writing – review & editing. JL: Writing – review & editing.
