# Supplementary material for: Hemoptysis caused by Parvimonas micra: case report and literature review
Source: Front Public Health. 2024 Feb 8;11:1307902. doi: 10.3389/fpubh.2023.1307902 (PMC10883377; doi:10.3389/fpubh.2023.1307902)
Supplement: Supplementary file 1 [file Data_Sheet_1.docx]

Supplementary Material

Hemoptysis Caused by Parvimonas micra: Case Report and literature review

**Axue Shao1†, Qingqing He 1†,**  **Xin Jiao1, Jianbo Liu 2***

^1^Guangzhou University of Chinese Medicine, Guangzhou, China

^2^ First Affiliated Hospital of Guangzhou University of Chinese Medicine, Guangzhou, China

* Correspondence: Jianbo Liu: [13538759071@163.com](mailto:13538759071@163.com)

## Supplementary Tables

**Supplementary Table 1.** Major test results at the time of the patient's admission

| Laboratory analysis | Level | Normal range |
| --- | --- | --- |
| CRP | 3.85mg/L | 0-8mg/L |
| WBC | 8.82E+9/L | 4.0-10.0E+9/L |
| RBC | 4.17E+12/L | 4.0-5.5E+12/L |
| HGB | 138g/L | 120-160g/L |
| PLT | 297E+9/L | 100-300E+9/L |
| NEU% | 71.60% | 50-70% |
| LYM% | 20.60% | 20-40% |
| CHOL | 3.64mmol/L | 2.6-5.2mmol/L |
| TG | 3.64mmol/L | 0.34-1.70mmol/L |
| HDL-C | 0.72mmol/L | >1.04mmol/L |
| LDL-C | 1.63mmol/L | <=3.37mmol/L |
| ALT | 10U/L | <=41U/L |
| AST | 15U/L | <=40U/L |
| ALB | 40.2g/L | 40-55g/L |
| A/G | 1.5 | 1.5-2.5 |
| Glu | 5.85mmol/L | 3.9-6.1mmol/L |
| UREA | 3.88mmol/L | 3.1-8.0mmol/L |
| CREA | 75ummol/L | 57-97ummol/L |
| UA | 466ummol/L | 208-428ummol/L |
| K | 3.03mmol/L | 3.5-5.3mmol/L |
| Na | 141.7mmol/L | 137-147mmol/L |
| Ca | 2.17mmol/L | 2.11-2.52mmol/L |
| HbA1c | 5.80% | <6% |

**Supplementary Table 2.** Identification of Parvimonas Micra in BALF using mNGS

| Genus | | | Species | | |
| --- | --- | --- | --- | --- | --- |
| Types | Latin name | Sequences | Latin name | Sequences | Relative abundance |
| G+ | Parvimonas | 583 | Parvimonas Micra | 583 | 2.90% |
| DNA | / | 41 | Human gammaherpesvirus 4 | 41 | 0.2% |

**Supplementary Table 3.** Research included in the literature review.
