# Supplementary material for: Hemoptysis caused by Parvimonas micra: case report and literature review
Source: Front Public Health. 2024 Feb 8;11:1307902. doi: 10.3389/fpubh.2023.1307902 (PMC10883377; doi:10.3389/fpubh.2023.1307902)
Supplement: Supplementary file 2 [file Data_Sheet_2.pdf]

Supplementary Material

Hemoptysis Caused by Parvimonas micra: Case Report and literature review

Axue Shao1†, Qingqing He 1†, Xin Jiao1, Jianbo Liu 2*

[1]Guangzhou University of Chinese Medicine, Guangzhou, China

[2] First Affiliated Hospital of Guangzhou University of Chinese Medicine, Guangzhou, China

* Correspondence: Jianbo Liu: 13538759071@163.com

Supplementary Figures

(A)

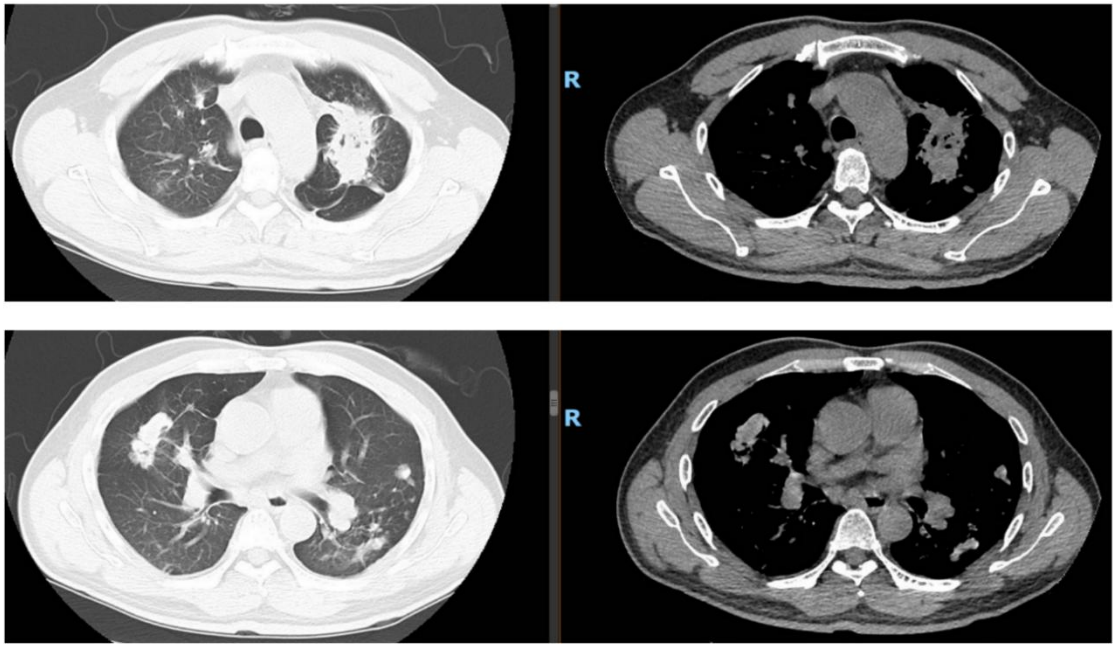

Supplementary Figure 1. The results of chest contrast-enhanced computed tomography scan demonstrated that scattered multiple nodules and mass-like shadows in both lungs mainly in both upper lungs, the largest one measuring about 58x46mm (left upper lung), irregularly shaped cavities and necrotic areas visible within.

(B)

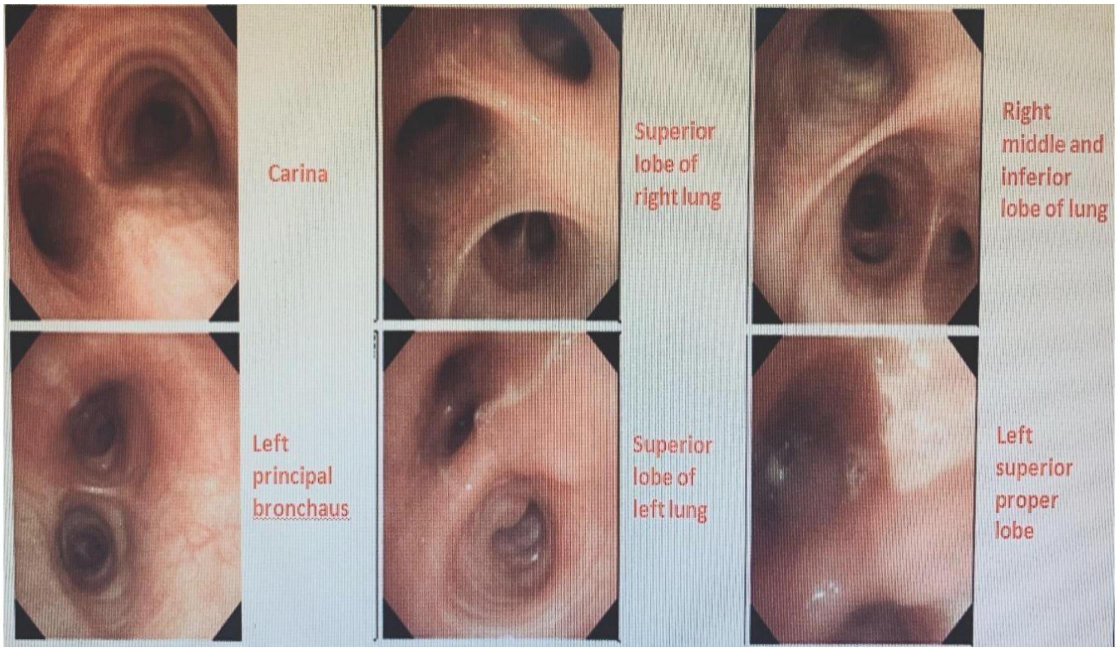

Supplementary Figure 2. The lesion of Bronchus on fiberoptic bronchoscopy

(C)

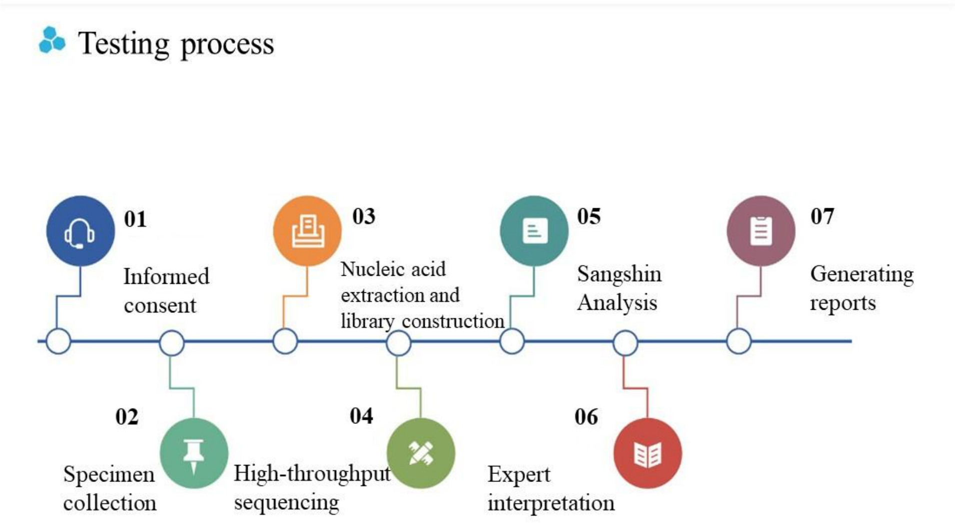

Supplementary Figure 3. mNGS Testing Process
